# Collectanea Medica, Consisting of Anecdotes, Facts, Extracts, Illustrations, Queries, Suggestions, &c.

**Published:** 1816-07

**Authors:** 


### COLLECTANEA MEDICA,

CONSISTING OF

### ANECDOTES, FACTS, EXTRACTS, ILLUSTRATIONS, QUERIES, SUGGESTIONS, &c.

RELATING TO THE

*History or the Art of Medicine, and the Auxiliary Sciences.*

Quicquid agunt medici,

Nostri farrago libelli.

IN a former Number, we gave some very interesting extracts from the examinations before the House of Commons on the subject of Insanity. Highly to the honour of the nation, the inquiry is still continued. We have been favoured with an early copy of the printed Minutes, some of the more important parts of which we shall extract for our readers.

*Sir Henry Halford, Bart.* called in, and examined.

Have the goodness to state to the committee, whether, in your opinion, medical advice is likely to be useful in cases of insane persons?—I have no doubt of it; it is most useful in the early stages of insanity; but it is useful also in the progress of the disease, particularly where it recurs in paroxysms; and it is occasionally useful in confirmed lunacy, though the good effect of it is less certain in the advanced stages of the disease. This, however, is analogous only to what is found to be the case in other distempers.

Can you form any probable conjecture of the proportion of cases in which medical advice might be useful, if applied early?—I cannot; it is necessary to know the circumstances of each case, and its history, before a physician can say to what degree medicine may probably be of use. I consider insanity to be connected with bodily indisposition, throughout its course, though this be less apparent in some cases than in others. It is obvious in the instances of females who become deranged after lying-in; this is, perhaps, the most remediable specimen of the disease; it is obvious also, in that modification of the malady which we see in females of a particular temperament, at a certain period of life, when they sometimes become melancholy; and it is striking in the cases of sailors, after a great sea-fight, where there had been previously great earnestness, much personal exertion, protracted watchfulness, and, after the conflict, an improvident indulgence in spirituous liquors. These combined causes produce great irritation of the brain, and derangement; but such patients generally get well. I remember to have seen at least twenty sailors in a state of derangement, in one house of reception of lunatics, after Lord Howe's victory on the 1st of June. I have stated that medicine is essential in the progress of insanity, more especially where the disease is wrought up into paroxysms, and recurs with violence in that form; in such paroxysms there is an appeal to the skill as well as to the humanity of the physician, beyond what arises in almost any other disease, for the body labours in this unhappy predicament until it is destroyed; I have seen several patients die in this painful manner. If medicine be less useful in the confirmed periods of insanity, it is as little so in the advanced stages of other chronic disorders. In cases of incapacity of the joints, with painful swellings upon them, from chalk-stones, after repeated fits of the gout, medicine has no effect upon these depositions; yet this is no argument against the use of medicine in the first attacks of gout, to prevent, if possible, such dismemberment and deformity. Again, in the instance of palsy, when a patient has lost the use of half his body; in this stage of his complaint medicine has very little sensible effect upon it; but if the patient be assisted in the earliest attack of his malady, whilst under apoplexy, which generally precedes palsy, not only may his life, possibly, be saved, but the paralytic symptoms prevented altogether, or at least considerably mitigated. But we have much to learn on the subject of mental derangement; and I am of opinion, that our knowledge of insanity has not kept pace with our knowledge of other distempers, from the habit we find established, of transferring patients under this malady, as soon as it has declared itself, to the care of persons who too frequently limit their attention to the mere personal security of their patients, without attempting to assist them by the resources of medicine. We want facts in the history of this disease, and if they are carefully recorded, under the observation of enlightened physicians, no doubt, they will sooner or later be collected in sufficient number, to admit of safe and useful inductions.

Have you any opinion as to this advantageous application of medicine, to cases of derangement not arising from temporary bodily causes, but from causes which may be classed as hereditary family ones. In the early developement of these cases, is not medicine advantageous for repelling the malady, if not for the purpose of radical cure?—Such cases are usually most difficult to cure; the pre-disposition in the frame being strong, external accidental causes act with more violence upon it, and more easily overset reason.

Does not the mixing patients who are outrageously mad with those who are quietly so, retard or lessen the probability of cure of the latter?—I have no experience of the treatment of insane persons collected together in numbers. My practice has been limited to individuals in private houses.

Can you say, whether in many cases of insanity, employment and occupation are not extremely likely to promote a cure?—Where the patient is quiet and well enough to be induced to employ himself, it contributes to his recovery.

Both

Both bodily and mental?—Yes; but it is not easy to prevail upon an insane person to occupy himself with such employment or amusement as you may set before him.

Would the attendance of one physician upon such a number as one hundred patients be sufficient, in case of his giving up his whole time to it?—Perhaps it might. But I should think it better to have two, three, or even four physicians, to attend such a number of patients, and not to limit their attendance to this collection of patients.

Do you think any inconvenience would arise from the attendance of two physicians on a county asylum or county hospital for insane persons, the same as upon hospitals for other disorders?—None; but, on the other hand, great convenience, for, besides the advantage of consultation in difficult cases, it would preclude that mystery in future in the treatment of insanity, which without having been of any service to the patients, has been the means of withholding from the profession at large, much valuable information on the nature of this disease.

Would it not be a great advantage to the patients, as well as facilitate the attainment of information with regard to the nature of the disease, to have two or more physicians attend one asylum for the reception of lunatics?—I have answered this question, I believe, already.

In institutions where there are fifty or one hundred patients of this sort, is it not absolutely necessary for their benefit that there should be a resident surgeon and apothecary?—I think it is absolutely necessary; for disorders of this description often demand instant attention and assistance.

And that his attendance should be confined to that institution? — Certainly.

Is it your opinion, that seclusion and restriction from the visitation of those places, are prejudicial to the patients, or necessary to be adopted?—A discreet visitation of these houses for the reception of insane patients, may be useful and proper; but it must be discreet.

Are you of opinion, that in every case of incipient insanity, the advice of a physician is most important?—I should think the advice of a physician, if it can be had, always important and useful in the beginning of insanity.

---

The following examination is particularly interesting, as containing the economy of a charity less known than Bethlem.

*Mr. Thomas Dunstan* called in, and examined.

What is your situation?—Master of Saint Luke's Hospital.

Is it the custom at Saint Luke's Hospital to confine any description of patients on straw only?—They lie upon blankets and straw, the wet patients.

And in the winter for several months together?—While they lie wet, we find it the most convenient, the urine runs off better than from any thing else, and we can shift it so much the oftener.

Have you ever sent any patients out of Saint Luke's with limbs contracted, in consequence of their having been thus confined in straw beds?—I do not immediately recollect; we have had them contracted rather; we always contrive to walk them about and keep people to lead them. I do not recollect that we have had any so contracted, that they could not walk.

Have you had any instances in Saint Luke's of patients destroying themselves?—That has been the case, but very rarely.

In those cases has a coroner's jury been invariably summoned? —Yes, where that has been the case, a jury has been summoned.

In every instance of suicide or sudden death, a coroner's jury has been summoned?—Yes; except they have died in a fit, and then their friends take them away.

Though that death may be entirely sudden?—Yes; if they die in a fit, that sometimes happens. I knew a man once die at his meals, there was no coroner's jury upon that, for it was deemed as a fit.

Has that fit ever been occasioned by the treatment?—Never, to my knowledge.

When the jury is summoned, as stated in cases of suicide, do you know how that jury is formed, whether by persons in the immediate neighbourhood, or serving the hospital with articles?—Not persons serving the hospital with articles; the constables summon the jury, and we never know who they will be till they come.

You never have interfered in the summoning of a jury?—Not in the least; we never knew who they were till they came.

Have you known instances of female patients being pregnant in the hospital?—We had one that was thought to be pregnant in the hospital; she was sent to me a good way up from the country; she came up in a broad-wheeled waggon, and it appeared that it was through the waggoner, as she came up; they laid it upon us, but the waggoner confessed it, and it turned out that she became so by the waggoner, as she came up.

Do you recollect a patient of the name of ——— being in Saint Luke's?—There was.

Was she pregnant while in the hospital?—She was, by one of the servants.

By which of the servants?—His name was Edward Dowding.

Was he dismissed for that offence?—He was.

Immediately?—I kept him a week or two to be certain. I was some time before I found it out, and then he was dismissed.

Do you know what became of him afterwards?—He now lives somewhere in Whitechapel, I believe; he married one of the servants after that.

He is not now connected with any house of reception?—None whatever, I believe.

What became of Miss ———?—She went to her father at Bethnal Green, I think.

Are you sure she did not go to Mr. Warburton's house?—I heard she was there several times.

Do you know whether she was there as a patient?—Yes, several times; but whether she went directly from me to that house I cannot tell; I thought they tried her at home first. I know they sent her there, but I thought it was not till afterwards. I think I heard that she lay-in there afterwards.

At the time she became pregnant by this man, was she confined in irons, her legs and wrists both confined?—She was not at all confined.

In a former examination you stated, that the men had no access to the women's side, except in cases of refractory patients; how then came Dowding to have access to Miss ——— ?—They carry up the beer and the provisions with the maids, and they are sent for occasionally, as I observed before.

Do you happen to know why Miss ——— was confined; was she mad?—Yes, she certainly was a vicious kind of young woman.

You have stated, that the apothecary pays his visits daily through the hospital; did you not send him into the country after a patient who made his escape from the hospital, and was he not at that time absent for a fortnight?—I am not certain how long he was absent, but he did go after a patient, I think not at that time.

While he was absent, did he not leave the key of his shop with one of the patients?—Not to my knowledge.

Can you state that he did not?—He did not, to my knowledge; I think I should have known it if he had, and I do not know that he did.

You do not know whether this person, with whom it is supposed he left the key, administered medicines to the patients?—I do not.

Do you know whether the medicines generally in use at the hospital, especially the bitters, are prepared by one of the patients?—No; there used to be a patient, who was in the hospital thirty years, who used to help Mr. Meadows, and he could make them very correctly indeed, and the man is there now; at times he is very poorly, and at other times very well.

Did you ever state to the governors that the apothecary was an unfit person to be in the situation?—No, I do not recollect that I did; I have thought him a little unsteady sometimes.

Not only on account of his neglect, but being a married man; is not that contrary to the rules?—It is.

He is a married man?—He is; I did not know that when he came.

You knew it afterwards?—Yes; he was on the point of going away when I knew of it.

Was not it known he visited his wife, and she came to him?—She was not there three times, and I looked upon her as his sister.

Is the apothecary employed by you in transacting your private business?—No.

In

In collecting rents or attending sales, or any thing of that sort?—Oh, no; I might send him to call for a little bill, or any thing of that sort.

Who superintends the hospital in your absence?—I and Mrs. Dunstan; we are never absent together, nor have been for above thirty years, and we sat down with that intention when we went to it.

How are the assistant-master and matron employed?—There is an assistant-master at this time employed under me.

Is the assistant-master's time taken up in any other avocation for your private advantage, or that of Mr. Dunstan of Broad-street, your son?—No, not that I know any thing of.

Is there generally a deficiency of two or three of the servants allowed by the hospital?—No; when one goes away, another comes.

How is the assistant-matron employed?—In attending to the linen.

Does the assistant-matron ever go out to take the care of ladies who may apply to you for a keeper?—Never; she has been there these thirty years, and never goes out at all.

Was she not with a lady at Halstead, in Essex, four or five months the last year?—No; I have had servants that when they have left me have gone out with people.

The question is, whether during the time she was assistant-matron of the hospital, she did not go out to take care of ladies for private emolument?—No, she did not.

You know that she was not with a lady at Halstead for four or five months the last year?—I do not know that she was, and I must have known of it if she had.

Did not a female lay herself upon the fire one evening, which occasioned her death, and was not an alarm given by a gentleman passing along Old-street, who saw her passing the windows in flames?—There was a woman who secreted herself, and the fire was raked, and she went and clapped her clothes all over the fire and cinders, and so she became on fire; the maid was just by her and laid her down immediately, and took her into her own room; she lived two or three months afterwards, but it was her death subsequently.

Had you an inquest upon her when she died?—No, the surgeon and doctor attended her.

Have not you several houses in which you accommodate patients to the number of three or four in each?—Not one, nor never had.

Do you mean to state that the private madhouse in Ivy-lane, Hoxton, did not belong to you, and that you did not receive the emoluments?—I am only the landlord, and never received any emoluments from it; our committee investigated that and were satisfied of it, that I only received the rent.

Do you mean to say, that you never have been at any time since you have been in the situation at Saint Luke's, the master or owner of any house for the reception of lunatics, and that you have never derived from any such house any emolument, as partner or otherwise?—I do.

Are you in the habit of recommending patients to Mr. Warburton?—I am.

For those recommendations do you receive any emolument or advantage whatever?—None whatever; Mr. Warburton has made my wife a present of a gown, and sometimes has given my son a few books.

You receive no pension or advantage?—No.

Nor no pecuniary allowance?—No.

Nor no profit or allowance in any way whatever?—No more than what I have mentioned.

Beyond that, no advantage has resulted to you from those recommendations?—No advantage whatever.

Have you now any patient confined in Saint Luke's, by means of an iron collar round his neck?—None; we never had.

You have stated that those patients who dirty themselves have clean blankets every night; are they fresh ones, or the same that have been washed during the day?—They are mostly fresh ones, or those that have been dried; we keep a fire to dry them.

Do you recollect a patient, in 1812, falling down the straw-shaft, and dislocated his thigh?—There was one who fell down, and hurt his hip-joint.

Was the dislocation ever reduced?—I am hardly able to say.

Did not the patient die within a fortnight? —I do not remember, but I think not.

How happens it there can be any doubt of the surgeon having applied his skill?—I do not recollect the particulars; I know there was one fell down, but I believe that he did not die, and I remember the surgeon was applied to.

You recollect the injury?—Yes.

But you do not recollect whether the complaint was treated, or not?—I cannot say particularly to that.

Is there no record kept in the hospital books of accidents happening?—None, that I know of.

If a person destroys himself, is that put down?—Yes; that is entered in the committee-book.

But not if they meet with any accident short of death?—No; a surgeon is sent for directly.

The entry of the death of this person would appear in the book? —Yes.

But not an accident, and of course not the date of it?—No.

Do you know that the practice of muffling exists!—No, never; sometimes the maid has tied a bit of sheet or something round the nose to dun the noise, to see whether it would quiet them; at other times put them by; some of them will bawl from Monday morning to Saturday night, and she has put a shutter up to prevent their disturbing others.

Do you know of the practice of forcing a patient, when they have have been unwilling to take food?—Yes; I have done it many score times.

Have you had any injury in consequence to any of the patients? —Not with me, in any instance whatever.

Do you recollect any such instances in your house?—No.

Have you ever employed any of the keepers about business not connected with the hospital?—Not particularly, that I know of; they have gone with an errand or a message, or something of that kind for me.

Was not one of the keepers named Thomas Flower employed daily for a month or two in looking after your son's shop?—Not that I know of; there was one Mr. Drury, an apothecary, used to go down to attend my son's shop for Mr. Rogers, and Mr. Rogers paid him for it.

Did you not permit a keeper named Thomas Flower to work at his business as a baker, for six successive months, to enable him to pay you a small debt?—No, he went away; and I, in fact, bought the lease of a baker's shop to put him in it, because he married one of the servants, and I lost £20 by him in consequence.

He was not employed as a baker while he was a keeper?—No, he was not. I beg pardon, there was a man who went to our baker's for some weeks; he used to go once or twice a week to make himself fit for the baking business; he married one of the servants in the house, and I let him go to fit himself for being a baker.

He used to go in the night?—Yes, part of the night.

Is your son's linen washed by the servants of the hospital?—Some little of it I do not deny; his frills and handkerchiefs, and such things.

*Mr. Thomas Dunston* again called in, and examined.

You recommend frequently, or advise, the families or friends of lunatics to take them to Warburton's?—Sometimes, if they ask me the question I do; never without.

Does that often happen?—Frequently.

Is it to one only of Warburton's houses?—Sometimes to one and sometimes to the other; I say they live very well, and are well taken care of, for any thing I know.

You recommend occasionally to either of the three?—Yes.

Whitmore House, Talbot's, the White House, and Rhodes's?—Yes.

When the patient proceeds to Warburton's for admission, what certificate of insanity does he take with him?—He takes a certificate from the person who sends him, and the medical man.

From the friend or friends who commit him, and from the medical man?—Yes.

What may be the meaning of the medical man?—Our apothecary mostly, mostly the apothecary of the house, sometimes the surgeon.

That the man has been admitted with a certificate into your house?—Yes.

Do

Do you in any way countersign the certificate for admission to Warburton's; does your name appear, or do your initials appear? —my initials have appeared.

Do your initials usually appear?—Sometimes they have, sometimes they have not.

What is the object of the insertion of this letter in the certificate?—I have no meaning, only that the keeper of the house may know they come from St. Luke's; I have no other meaning in it.

The persons you refer to have been already in St. Luke's?—Yes, and discharged uncured at the expiration of twelve months and six days.

What may be the advantage of Mr. Warburton, the keeper, knowing that those patients do come from Saint Luke's?—I do it upon these grounds, that they have not much to spare, that they are mostly poor, and to be as moderate with them as possible; I have no other meaning.

The meaning of this letter is to prove the poverty of the patients?—Yes, he came from Saint Luke's, and it is well known that those who go from us have nothing to spare.

Are you now speaking of pauper patients?—Sometimes they are poor people, more needy than paupers on many occasions; and I have sometimes written at the bottom, begging them to be as moderate in their charges as possible, for that the friends could not afford to pay much.

No governor can recommend a patient to Saint Luke's, unless he is in poor circumstances?—He cannot.

A certificate of a patient going from Saint Luke's to Warburton's, proves that he is poor?—It does; when it has been suggested to me, from knowing the distresses of the family, I have begged them not to take more than twelve or thirteen shillings a week, for that the family could not afford it, and I have saved scores of people in my life-time pounds and pounds by it.

The insanity must be proved, and the qualification of poverty must appear to the Governors of your hospital?—Yes.

How does that appear?—From the certificate of the churchwardens and overseers, and the minister of the parish, where they have resided; and the gentlemen of the committee always examine it strictly when they come, and if they find they have sufficient to support themselves, they do not admit them.

Are many of these parish paupers as well as poor parishioners? —Many of them.

What is the weekly charge?—I believe the paupers pay about ten or eleven shillings a week: from ten to eleven shillings I think is the usual charge, and the friends pay something more.

Every patient in your house, male and female, lies alone?—Yes, all in separate beds, but in some rooms they have two or three beds; and I have in some of the rooms put two bedsteads in a room on purpose for safety; for they will try all schemes to make away with themselves, but where there is another in company they will not do it. The gentlemen have asked me why I allow two beds in a room, and I have given that as my reason, and that it is not for any other reason whatever.

Have you ever, in the course of your practice, known an instance of a lunatic making an attempt upon his own life in the presence of any other person?—I do not known that ever I did.

Do not many of your patients dine together?—Yes.

Do you trust them with knives and forks?—We never trust them with any; the meat is cut thin and laid upon trenchers, for fear of any thing of that kind occurring.

You have repeatedly assured the committee, that you have no interest whatever in either of Warburton's houses?—Nor no others; I have sent servants out who have lived with me and gone out, and have never received a shilling in my life.

What number of patients have you in Saint Luke's now?—I think 264; the number is very low now to what it has been; we have had 340 or 350 waiting for several years.

There is no candidate for admission now?—No.

Of those 264, can you inform the committee how many are under personal restraint; how many are chained or in straight waistcoats?—I cannot exactly say; there may be sixteen or eighteen, or there may be two or three more. Many of them are in a poor lost low state, and tearing their things to pieces; to prevent this, we are obliged to keep them in waistcoats in the day, for no other reason but that.

Of those sixteen or eighteen under personal restraint, what proportion are without clothes?—None without clothes; they are got up every day, and their clothes put on, and the waistcoats over.

Miss ——— referred to in your evidence yesterday, went from Saint Luke's?—She was discharged well and went home to her friends. I have ascertained, that since I attended the committee yesterday, she relapsed and came back again; it was the second time she was with us she became pregnant; she was again discharged well.

Do you know any thing of her having gone to Warburton's afterwards?—Only by hearsay.

She did not go with any certificate from you or the surgeon?—No, she could not, for she was discharged well; there was no certificate at all.

What was her situation in life?—Her grandfather was a stockbroker, and her father was a stockbroker; and I heard her own father went beyond his mark in speculations or something, and was written up in the hall, and dismissed, and did not go there afterwards, and the grandfather followed the business; he was a very respectable old gentleman.

Do you consider her as having been a very poor woman?—I think she was, for she had nothing but what she got from her grandfather; and her grandfather kept the son and his family.

Have you been able to ascertain any further circumstances respecting the intercourse between the keeper Dowding and Miss ———?—The father and grandfather and myself talked to her many many times, I suppose for ten days, desiring to know how it happened, and who it was; she positively declared to me over and over again, that it was her own brother, I never saw any person stand to any thing as she did; at last I persuaded her to tell me, and she did; she told me it was Edward Dowding, and that it happened in the dusk of the evening, when he went up with the beer, behind the door; there were two patients in the room at the same time, and never saw it; and he told me the same himself, when I found it out.

Did the keeper when taxed confess it?—On being a good deal talked to, he confessed it.

She having charged him with having got her with child, and he confessing he was the father of the child, you dismissed him?—Yes, I did, as soon as I could find out the facts.

Is that the only case in which a female patient has been got with child in Saint Luke's?—I never remember one before nor since.

When you found her with child, did she lie-in in the hospital?—No; she went home to her friends before that time.

Had she come in as a parish pauper?—No; her grandfather put her there, and her own father was there I believe at the same time; he was a patient in the house, part of the same time that she was.

She charged her brother with having been the father of the child she bore at that time?—Yes.

Was the brother in the house at the time then?—No, the grandfather, the father, and the brother, all came to me in the parlour, and we taxed her altogether, and she bore us out before his face that he was the father.

Had he been to see her?—No, he had never been near the house, he could not have done it in our house, for he never came to see her, therefore I was sure it must be untrue.

No male keeper has access to any of the insane women, but at meal times?—No, unless they are wanted; if a patient is very obstreperous, so that they cannot manage her, they send for a man.

How do they get in, when they are sent for?—The same key unlocks all the gates. I lock them up at night, so that they cannot get into the gallery without my leave.

In the day-time, the same key will admit them?—Yes, but they do not go.

How do you know that they do not go?—I should be sure to hear of it, there are no secrets amongst them.

Does not each male keeper's key give him, during the day, access to the female wards?—They have a key that would give them access, or we should be very much at a loss; if any thing happened, we should not be able to get there in time to save any mischief, if it was not for that.

You say that no knives and forks are allowed, but that the food is cut for them, and served to them in trenchers?—Yes.

How is it with patients that wear a straight waistcoat the whole of the day?—That is cut by the servants in their room, and they are fed with it.

The servant applies it to the mouth of each patient? —Yes.

And the same with respect to what the patient drinks?—Just the same, there are several of the patients that are out of the waistcoat, that will not eat or drink unless they are crammed, they will not take it themselves; they will not resist it if we feed them, but they will not take it themselves; that is the case with two at this very time: several of those in straight waistcoats are undone while they are at dinner; some we can trust, and some we cannot.

Your keepers have, in consequence of that, some additional labour?—Yes.

What may be your number of keepers at this time?—Only one to each gallery; there are always some patients better than others, and the best doctor they have is employment; if we can get them to assist the maid, that does them a great deal of good; the servants are obliged to have some of the patients to assist them.

How long have you been employed in this line?—I believe about four-and-forty years.

How long have you been in the situation of master of Saint Luke's Hospital?—I think between three and four-and-thirty years.

You as master, and your wife as matron?—Yes.

With what salary did you commence the office of master?—I think at first we had but sixty pounds a year between us, there were but thirty or forty patients at that time.

This was in the old hospital?—It was; the gentlemen then gave us some gratuity; the committee have many times told us that we were not half paid; we have never gone out of the house together.

You have occasionally received gratuities from the general committee, in consideration of their approbation of your conduct?—Yes, I have, and that very lately.

At the last court, did you receive any gratuity?—It was passed by the court; I have not yet received it; they voted me fifty pounds.

In cases of sudden death at the hospital, you have stated that there are only particular cases where the coroner sits?—Yes, in cases of suicide.

Has that been the practice ever since you have known the hospital?—Yes.

Did you ever hear a reason for not summoning the jury in cases of sudden death as well as felo-de-se?—No, I never did; there was one coroner when I first came to the house; there was a coroner, I think the gentleman's name was Beach, that had given Mr. Mansfield, the first master, a general warrant whenever any thing happened to bury the people when any accident happened; that is forty years ago, I dare say.

Had you any general warrant from the coroner?—Never.

You never had any coroner's inquest except in cases of suicide? —Only in cases of suicide.

When

When a patient dies suddenly, and has not been seen by a medical man?—We have had a case of a patient who has died in a fit, and a medical man has been called in.

Is it the practice where a patient dies in a fit, and is not seen by a medical man, to have a coroner's inquest?—I never remember an instance of that.

Is there not a resident apothecary in the house?—Constantly.

Does he see the patients every day?—Regularly every day.

If a patient were to die suddenly in the night, without having been seen by a medical man, should you call for the coroner's jury?—There was one man who was taken in a fit, while the servant was feeding him, and they took him to bed; and the apothecary was sent for to him and saw him, and he died after that.

If a medical man, on being called to the relief of a dying man, found the patient in question dead, would you in that case send to the coroner?—Yes, if he was dead I should; but I never remember but this instance, and then the apothecary had seen him, and we did not.

If one of the patients knocked the other on the head, would you in that case send to the coroner?—Yes, directly; but I have not had any experience of that.

No man ever died so suddenly, as not to have seen some medical man of the establishment, before he drew his last breath?—I do not recollect one instance of the kind.

For some years Dr. Simmonds was the superintending physician at Saint Luke's Hospital?—He was for more than thirty years.

Did you know him well?—I did.

Did you ever converse with him respecting the management of Saint Luke's?—I have many times.

Did you hear him say any thing on the subject of frequent visitations of Saint Luke's Hospital, by governors or others?—I have many times. He thought the less they were visited the better; that it irritated and disturbed them much, and made them much worse than they otherwise would be.

Their seeing any body?—Yes.

Do you know any thing of the Doctor's interference with the committee, desiring them not to visit too frequently?—I think I recollect his applying to the committee, and wishing it not to be.

You do not recollect how that arose?—I believe it was from a disturbance in the house among the patients, on visiting days, that they used to be mixed men and women together. They were very bad, and the people would, out of humanity and tenderness, bring them things to eat, and they were then ill for two or three days. The doctor interfered, and it was then altered to once a fortnight.

Do you recollect any interference on the part of Doctor Simmonds respecting the committee visiting?—Yes, I think I recollect his interfering and saying, that if they visited, it made the patients more insolent and impudent than they otherwise would be, as they have been, I am sure.

Do you not think, that if there was a separation of the lunatics into into smaller classes, the evil arising from visiting would be very much diminished?—I think it might.

Do you remember Mr. and Mrs. Ford, who were assistant-master and matron of the hospital?—Yes.

When did they come?—As much as two or three years ago; they were there six or seven weeks, and went home one week or ten days.

Did they come recommended by the committee?—No, only by my seeing the man at the house, and understanding that he kept a house for the reception of people at Maidstone, or some place in Kent; and he came in and was there for a time; but we went to the committee at Batson's. I do not know of whom the committee consisted, but the treasurer and six or eight gentlemen. They saw Mr. Ford, and they called me in and asked me about Mr. Ford; and all that I said was this—that I could not say so much as I wished to do. And one of the gentlemen said, "Mr. Dunstan, we are of your opinion; we do not think he is a man that will suit us." And I said, I was afraid not; indeed, I was sure not; for I did not suppose him to be so unwieldy a man as he was; he was a lusty man. But his wife told our apothecary, Mr. Drury, at that time, that he would not wash his face for three months together, if she was not to stand and do it for him.

To what should you attribute the falling off in the number of patients in Saint Luke's Hospital at present?—To the institutions in the country, I should think.

How many vacancies have you in your house at present?—Thirty-six. I have never known such a thing for years.

Have you been able to ascertain who was the female who attended a lady at Halstead in Essex, four or five months last year, and whether she was an assistant-matron in Saint Luke's?—I have ascertained who the person was, and that she never was a servant in Saint Luke's Hospital; her name is Tow.

We have given this long extract that we might do justice to this most respectable establishment. The account is also sufficient to show, as we have before hinted, that madness does not encrease in proportion to our population, or other causes. It also offers us a very important hint on the means of preventing suicide in maniacs.

